# The application of cell surface markers to demarcate distinct human pluripotent states

**DOI:** 10.1016/j.yexcr.2019.111749

**Published:** 2020-02-01

**Authors:** Jacob Goodwin, Andrew L. Laslett, Peter J. Rugg-Gunn

**Affiliations:** a CSIRO Manufacturing, Research Way, Clayton, VIC 3168, Australia; b Australian Regenerative Medicine Institute, Monash University, Wellington Road, Clayton, VIC 3800, Australia; c Epigenetics Programme, The Babraham Institute, Cambridge, UK; d Wellcome-MRC Cambridge Stem Cell Institute, University of Cambridge, Cambridge, UK

**Keywords:** Antibodies, Cell surface markers, Immunophenotyping, Naïve, Primed, Pluripotent stem cells, Reprogramming, 2i, dual MEK and GSK3 inhibition, 3i, MEK GSK3 and BMP inhibition, 5iLA(F), MEK GSK3 ROCK SRC and RAF inhibitors supplemented with LIF and Activin A with optional FGF2 E8, a commercially available media, FGF2, fibroblast growth factor 2, hPSC, human pluripotent stem cells, KSR, KnockOut™ Serum Replacement, LIF, leukaemia inhibitory factor, NHSM, naïve human stem cell medium, PGXL, MEK tankyrase and PKC inhibitors with LIF, RSeT™, a commercially available media, t2iLGö, titrated 2i with LIF and PKC inhibitor

## Abstract

Recent advances in human pluripotent stem cell (hPSC) research have uncovered different subpopulations within stem cell cultures and have captured a range of pluripotent states that hold distinct molecular and functional properties. At the two ends of the pluripotency spectrum are naïve and primed hPSC, whereby naïve hPSC grown in stringent conditions recapitulate features of the preimplantation human embryo, and the conventionally grown primed hPSC align closer to the early postimplantation embryo. Investigating these cell types will help to define the mechanisms that control early development and should provide new insights into stem cell properties such as cell identity, differentiation and reprogramming. Monitoring cell surface marker expression provides a valuable approach to resolve complex cell populations, to directly compare between cell types, and to isolate viable cells for functional experiments. This review discusses the discovery and applications of cell surface markers to study human pluripotent cell types with a particular focus on the transitions between naïve and primed states. Highlighted areas for future study include the potential functions for the identified cell surface proteins in pluripotency, the production of new high-quality monoclonal antibodies to naïve-specific protein epitopes and the use of cell surface markers to characterise subpopulations within pluripotent states.

## 1 Cell surface markers to investigate cell phenotype and function

Identifying and isolating specific cell types at single cell resolution is a major challenge across many areas of biology. This long-standing challenge is still relevant today as we gain an appreciation of the heterogeneity within cell populations and as we seek new ways to untangle this complexity. Cell surface markers that are recognised by antibodies have been at the forefront of cell phenotyping for many years [1,2]. This approach can help to resolve complex cell populations and importantly can also isolate viable target cell types for downstream functional studies.

Although applicable to all cell types, cell surface marker phenotyping has been used particularly effectively to classify cells within the haematopoietic lineage. The early adoption in this system of using antibodies for cell surface marker phenotyping was facilitated by the establishment of the ‘Cluster of Differentiation’ (CD) nomenclature in 1982 [3]. The nomenclature resulted from the evaluation of cell surface epitopes that are recognised by monoclonal antibodies, and the development of a framework into which additional markers can be added as they are discovered. This international initiative provided standardisation and reagent validation, and this effort has been instrumental in enabling an accurate description of cell phenotypes [4]. The CD nomenclature contains a broad spectrum of plasma membrane-localised epitopes including receptors, ligands and modifications, and, in addition, there are other widely used cell surface markers that are not currently designated as CD antigens. Cell surface marker expression changes depending on cell type and stage of differentiation, and antibodies that detect these markers are used widely in research, and for the diagnosis, monitoring and treatment of disease. Identifying informative markers and suitable antibodies remains challenging and is a bottleneck in many areas of biology.

Antibodies that detect cell surface markers are analysed predominantly by flow and mass cytometry, imaging and biochemical methods, thereby providing a quantitative and multi-parameter measurement of cell phenotype. When antibody-labelled cells are combined with flow cytometry-based cell sorting or separation using magnetic beads then target cell types can be isolated with high purity and sensitivity, or unwanted cells can be depleted from a population. Cell surface markers, therefore, provide a direct and objective approach for identifying and enriching target cell types.

## 2 Assessing human pluripotent stem cell heterogeneity using cell surface markers

Pluripotency is a property that describes the ability of a cell to differentiate into any of the tissue types present throughout development and into adulthood [5]. Human pluripotent stem cells (hPSC) are derived from early stage embryos [6] or are reprogrammed from somatic cells through the overexpression of OCT4, SOX2, KLF4 and c-MYC or similar transcription factors [7,8]. The ability to specialise into all cell types endows hPSC with exciting promise for applications in regenerative medicine and to study disease. To achieve this, characterising the pluripotent cell types and tracking the changes in their cell state during differentiation and reprogramming requires accurate and robust methods. Monitoring cell surface marker expression using antibodies is one such method that has been applied successfully to hPSC to provide a standardised measure of cell status when comparing between culture conditions [9], for optimising protocols that promote targeted differentiation including the isolation of target cell types [[10], [11], [12]], and in aiding the discovery of the molecular mechanisms that underpin pluripotency and reprogramming [13].

Several valuable and well-used cell surface markers were identified in undifferentiated human embryonic stem and carcinoma cells and provide an accurate readout of cell state. Commonly used cell surface markers include the globoseries glycolipid antigens SSEA3 and SSEA4 [14,15] and keratan sulphate related antigens TRA-1-60, TRA-1-81 and GCTM-2 [[16], [17], [18]]. As hPSC exit the undifferentiated state and initiate differentiation, there is a switch in glycolipid synthesis from globoseries to lactoseries and ganglioseries structures [19], which results in the loss of these cell surface antigens. Antibodies that measure this panel of epitopes have been instrumental to quantitatively compare between different cell lines and conditions [9], including hPSC derived from embryos and reprogrammed from somatic cells. These markers are also used to purify hPSC to relative uniformity [20,21] and to eliminate residual undifferentiated cells from differentiated cell populations as a strategy to improve the safety of hPSC-based therapies [22,23].

Combinations of cell surface markers and antibodies can fractionate hPSC cultures into discrete cell subpopulations that have distinct molecular and functional properties. Cells marked by the reduced expression of the cell surface markers GCTM-2 and CD9 have initiated the early stages of cell differentiation [[24], [25], [26]]. In contrast, cells that express high GCTM-2 and CD9 transcribe high levels of pluripotency genes and yield teratomas following in vivo transplantation. Interestingly, a very recent study reported a population of cells (comprising ~15% of the total), termed pluripotent founder cells, that are identified by cell surface NCAM (CD325) expression [27]. The study provides evidence that these cells are responsible for sustaining hPSC cultures. The majority of NCAM-positive cells express SSEA3 and TRA-1-60, and the cells have higher colony initiating capability compared to NCAM-negative cells. This effect was also observed when the hPSC were first pre-sorted for SSEA3-high expression: the NCAM-positive subpopulation still showed a higher efficiency of forming colonies. NCAM knockdown had no effect on colony initiating capability, demonstrating that NCAM is an informative marker but apparently not functional in this context. Several other cell surface markers were initially tested but did not define a discrete population of hPSC. Notably, subsequent analysis showed that several alternative cell surface markers are transcriptionally expressed preferentially in the NCAM-positive population, including several genes within the non-canonical Wnt pathway such as Syndecans 2 and 4, and other genes such as Integrins 1, 3 and 5. These cell surface proteins might be useful additional markers of pluripotent founder cells and it will be important to test whether they are expressed and informative. In addition, the capture of pluripotent founder cells raises interesting questions about the potential overlap between the NCAM-positive cells and the GCTM-2/CD9-high cells. The two sub-populations share certain features such as high colony forming capability, localisation to the colony boundaries and the expression of endoderm-associated genes, and an important next step is to see if they represent similar or distinct fractions. Taken together, these studies highlight how flow cytometry-based sorting for cell surface marker expression allows the fractionation of hPSC cultures into subsets of cells that hold different functional and molecular properties.

## 3 Investigating the expanding range of human pluripotent states

Several years ago, defined culture conditions were identified that can hold mouse pluripotent stem cells in two different states that are termed naïve and primed [[28], [29], [30]]. The two states are functionally and molecularly distinct and reflect their discrete developmental identities [31,32]. It was clear from this work that all hPSC lines at the time closely resembled the primed state of mouse pluripotent stem cells [33]. As mouse naïve pluripotent stem cells were reported to have certain advantages over their primed counterparts, this observation galvanised the search for conditions that could support the growth of an equivalent human cell type. The advantages described include high single cell cloning efficiency, a more uniform cell population, and the ability to produce high-efficiency chimeric animals indicating that naïve cells may represent an unbiased pluripotent cell [28,29,34]. The idea that alternative states of human pluripotency could exist was also supported by single cell transcriptional data that profiled human embryos at different stages throughout early development and implantation [35,36]. The data show that cells have distinct gene expression patterns at each of the collected stages and that transcriptional states exist in early human development that differ from all hPSC lines that were available at the time [[35], [36], [37]].

In the short period of time since then, several cell culture conditions have been reported that can sustain different states of human pluripotency. These new cell types include naïve hPSC grown in conditions such as t2iLGö, PXGL and 5iLA(F) that recapitulate features of the preimplantation human embryo, and that are distinct from the existing primed or conventional hPSC that align closer to the early postimplantation embryo [[38], [39], [40], [41]]. Other studies using alternative, less stringent conditions such as NHSM, RSeT™ and 3iL have proposed cell types that might fall somewhere within a range of developmental states and these cells have overall transcriptional profiles that are more similar to primed hPSC [[42], [43], [44], [45], [46], [47], [48], [49]]. Naïve hPSC grow in domed colonies rather than as flat monolayers, and have molecular properties such as global hypomethylation, reactivation of X-chromosomes in female cell lines and the induction of preimplantation transcriptional programmes [39,[50], [51], [52], [53], [54]]. Naïve hPSC, therefore, provide a much sought after cell model for studying pluripotency in preimplantation human development. Some of the other anticipated properties based on expectations from mouse, such as the ability to form blastocyst chimeras, have not so far been borne out by the data and will need to be tested further [55]. Several reports have shown that the differentiation capacity of hPSC depends on their starting cell state, which has important implications for producing tissues that are required for regenerative medicine and disease modelling [[56], [57], [58]]. For example, a recent article described that several, but not all, features of naïve hPSC are induced in primed hPSC in response to reducing the lipid concentration of the culture media [57]. This shift in pluripotent cell phenotype provides a starting cell type that can generate neural cells at a higher efficiency compared to primed hPSC in conventional growth conditions [57]. In addition, a recent report described the generation of human ‘expanded potential stem cells’' that showed some capacity for embryonic and extraembryonic cell differentiation [59]. Different growth conditions can therefore stabilise alternative states of human pluripotency, with naïve and primed hPSC at the two ends of the spectrum.

## 4 Cell surface markers for studying human naïve and primed pluripotent states

To help resolve pluripotent cell types, several molecular criteria have been put forward to try and benchmark the properties of hPSC against expectations drawn from the primate embryo [37,55,60]. The measurement of cell surface markers can provide an additional, robust and simple approach to compare directly between cell types [61,62]. Cell surface markers also have valuable roles in isolating viable target cell types from within a highly heterogeneous cell population that is a common feature when converting cells into a naïve state of pluripotency. Although naïve hPSC can be obtained directly from human preimplantation embryos, more often these cells are generated by reprogramming primed hPSC or somatic cells to a naïve state by exposing them to conditions that induce their cell state conversion. The advantages of reprogramming to a naïve state over embryo derivation include the ability to perform functional studies, the availability of greater cell numbers, and avoiding the restrictions associated with human embryo research. However, as with most reprogramming systems, reprogramming efficiency is low, the process takes a long time (up to 30 days in some reports) and produces a high level of cell heterogeneity particularly in the intermediate stages of reprogramming. As a result, we lack accurate characterisation, and a molecular understanding of the changes that occur during hPSC state transitions is still relatively unknown [[63], [64], [65], [66], [67]].

Several recent reports have described suitable cell surface markers and have begun to use these markers to track and study nascent naïve hPSC as they are formed during cell reprogramming. These studies have identified cell surface markers using different approaches, including by analysing expression levels in naïve and primed hPSC, as well as incorporating data from different stages of early human development and comparison with other mammalian species (reviewed in Refs. [61,62]). A common method to assess these differences is based on transcriptional datasets. These data can be analysed by unsupervised clustering of naïve and primed hPSC transcriptomes together with different stages of development to identify informative markers of pluripotent cell types. One of the potential pitfalls of using transcriptional methods is due to the frequent discordance between transcript and protein levels as a result of post-translational processing [68]. Interestingly, a recent study in mouse pluripotent stem cells showed that naïve cells have a stronger correlation between transcript and protein levels compared to primed cells [69]. This indicates that a number of potential new markers of naïve and primed pluripotency may be missed when using transcript information only.

Another approach identified cell surface markers using commercially available antibody libraries that contain several hundred antibodies recognising cell surface epitopes [64]. The use of a direct antibody-based approach has a number of benefits over other screening methods including the measurement of protein expression rather than transcript levels. Antibodies can also detect modified antigens such as glycoproteins that cannot be examined transcriptionally, as well as the direct compatibility with technologies such as flow cytometry. A downside to using antibody screens is the reliance on commercially available and high-quality reagents. Future studies could incorporate protein expression datasets to identify additional cell surface markers that can distinguish between naïve and primed hPSC.

Through these various methods, recent studies have identified a number of cell surface antigens (or proteins predicted to localise at the plasma membrane) including many that are yet to be fully characterised. Here, we discuss what is known about the current cohort of cell surface markers.

### 4.1 Primed-specific cell surface markers

Several cell surface markers are detected at higher levels in primed compared to naïve hPSC (Table 1). Surprisingly, given the long standing association with human pluripotent cells, SSEA3 and SSEA4 fall into this category. Both markers are uniformly high in primed hPSC but, in contrast, SSEA3 expression is low in 5iLA(F) and t2iLGö naïve hPSC [64,65]. Cells induced by less stringent formulations such as NHSM and RSeT™ cells retain high SSEA3 signal [65]. Expanded potential stem cells express SSEA4, TRA-1-60 and TRA-1-80 [59]. Interestingly, SSEA4 is typically heterogeneously expressed in established naïve cultures [51,64,65]. Cells within the SSEA4-low fraction express the highest levels of naïve-associated genes, whereas the SSEA4-positive population has a transcriptional state somewhere intermediate between naïve and primed hPSC [51]. The reduction in SSEA3 and SSEA4 levels could perhaps indicate that there is less glycolipid synthesis in naïve compared to primed hPSC.

**Table 1:** A summary of primed-specific cell surface markers in hPSC.

Surface antigen	Protein name	Gene symbol	Protein function	Cell culture conditions and references	
				High surface antigen expression	Low surface antigen expression
SSEA3	N/A	N/A (glycoprotein)	Unknown	KSR + FGF2 [64] E8; RSeT; NHSM [65]	5iLA(F); t2iLGö [64,65]
SSEA4	N/A	N/A (glycoprotein)	Unknown	KSR + FGF2 [51,64] E8; RSeT, NHSM [65]	5iLA(F) [51] 5iLA(F); t2iLGö [64,65]
CD24	Signal transducer CD24	*CD24*	Sialoglycoprotein; potential signal transducer	KSR + FGF2 [64] E8 [65,71] NutriStem XF [70]	5iLA(F); t2iLGö [64,65] RSeT [64] NHSM [70] PXGL [71]
CD57	Galactosyl-galactosylxylosyl-protein 3-beta-glucuronosyl-transferase 1	*B3GAT1*	Transfers carbohydrate epitopes onto glycoproteins	KSR + FGF2; RSeT [64]	5iLA(F); t2iLGö [64]
CD90	Thy-1 membrane glycoprotein	*THY1*	Glycoprotein; potential roles in cell adhesion and communication	KSR + FGF2; RSeT [64]	5iLA(F); t2iLGö [64]
NLGN4X	Neuroligin-4, X-linked	*NLGN4X*	Carboxylesterase/lipase; potential role in cell-cell interactions	E8; RSeT, NHSM [65] KSR + FGF2; E8 [72]	5iLA(F); t2iLGö [65] 5iLA(F); RSeT; NHSM [72]
PCDH1	Protocadherin-1	*PCDH1*	Potential role in cell adhesion and interactions	KSR + FGF2; E8 [72]	5iLA(F); RSeT; NHSM [72]
ADGRG2	Adhesion G-protein coupled receptor G2	*ADGRG2*	Orphan receptor	KSR + FGF2; E8 [72]	5iLA(F); RSeT; NHSM [72]
CDH3	Cadherin-3	*CDH3*	Calcium-dependent cell adhesion protein	KSR + FGF2; E8 [72]	5iLA(F); RSeT; NHSM [72]

A second primed-specific marker is CD24. CD24 expression is higher in primed compared to naïve hPSC that are maintained in t2iLGö, PXGL, 5iLA(F), NHSM and RSeT™ conditions [64,65,70,71]. In reprogramming experiments, CD24 marks quite a broad population of cells, and can be helpful when used in combination with a naïve hPSC marker to discriminate cells that are at an advanced stage of reprogramming [64,70,71]. CD24 is a sialoglycoprotein that is expressed on mature immune cells and modulates growth and differentiation signals to these cells. Whether CD24 is functional in primed hPSC or during the early stages of reprogramming is currently unknown.

An antibody screen found that CD57 expression is high in primed cells and low in t2iLGö and 5iLA(F) cultured naïve hPSC [64]. Cells that are propagated in RSeT™ media retain CD57 expression, suggesting that the reduction of CD57 signal defines a narrower range of cells as compared to CD24 expression [64]. CD57, therefore, provides a helpful readout to distinguish cell types when used in combination with naïve hPSC markers. CD57, encoded by B3GAT1, is a member of the glucuronyltransferase family and transfers carbohydrate epitopes onto glycoproteins. No function for CD57 in primed pluripotency has been reported.

A fourth cell surface protein is CD90 (encoded by *THY1*), which is involved in cell adhesion and cell communication in numerous cell types, particularly in cells of the immune and nervous systems. CD90 expression is high in primed hPSC and is reduced rapidly at the onset of reprogramming towards a naïve state [64]. *THY1* is a predicted FGF signalling target gene and so the switch in culture conditions from FGF-activation to FGF-inhibition at the start of reprogramming is likely to trigger the rapid downregulation in CD90 expression. Consequently, CD90 is less useful as an individual marker as it probably reads out signalling responses rather than as an accurate indicator of cell state change.

Another informative cell surface marker is NLGN4X, which is a member of the type-B carboxylesterase/lipase protein family and is implicated in mediating cell-cell interactions. A monoclonal antibody raised against this protein was reactive to ~95% primed hPSC and ~30–40% naïve hPSC cultured in 5iLA(F) and t2iLGö conditions [65,72]. As the NLGN4X signal is higher in primed compared to naïve hPSC when measured by flow cytometry, this antibody can be used to help discriminate between the two cell types [65]. Interestingly, human somatic cells that were reprogrammed in NHSM conditions were NLGN4X-low, but retained expression of the primed markers CD24, SSEA4 and F11R [65]. The precise timing of NLGN4X expression changes during reprogramming is unknown, although this observation suggests that NLGN4X is downregulated fairly early on and occurs before the other changes that mark the entry of cells into naïve pluripotency.

Three other cell surface markers and monoclonal antibodies were identified that are uniformly expressed in primed hPSC (>80%) and have reduced levels in 5iLA(F) naïve hPSC with reactivity to ~30%–80% cells, depending on the cell line [72]. The proteins are PCDH1, ADGRG2 (also known as GPR64) and CDH3, and they have functions in other cell types that are associated with cell adhesion and communication. The expression dynamics of these three markers during naïve hPSC reprogramming is not known.

Other reported cell surface proteins that are higher in primed compared to naïve hPSC (maintained in t2iLGö) include the NOTCH family of receptors and the NOTCH ligand JAGGED2 [64]. Although the receptors are present, the NOTCH signalling pathway is thought to be inactive in primed hPSC, and is then activated upon receiving differentiation cues and is required for multi-lineage cell differentiation [73]. One possibility is that the NOTCH pathway is poised for activation in primed hPSC to ensure effective cell differentiation, but that this role is not required in naïve hPSC as they lack features of lineage-priming. Of note, this developmental stage-specific expression pattern is similar to mouse pluripotent stem cells where Notch receptors are expressed in primed cells but to a lesser extent in embryonic stem cells [74]. The utility of NOTCH receptors and their antibodies as informative cell surface markers to distinguish between naïve and primed hPSC is currently untested.

### 4.2 Naïve-specific cell surface markers

The discovery of cell surface markers that are expressed by naïve hPSC enables the positive identification of naïve hPSC and for isolating these cells after their reprogramming. Several cell surface markers that are expressed in naïve hPSC have been reported (Table 2), and are used most effectively in combination with primed markers such as CD24, CD57 or SSEA4.

**Table 2:** A summary of naïve-specific cell surface markers in hPSC.

Surface antigen	Protein name	Gene symbol	Protein function	Cell culture conditions and references	
				High surface antigen expression	Low surface antigen expression
CD75	N/A	N/A (glycoprotein)	Unknown	5iLA(F); t2iLGö [64] PXGL [71]	KSR + FGF2; RSeT [64] E8 [71]
CD130	Interleukin-6 receptor subunit beta	*IL6ST*	Cytokine signal transduction	3iL [44] 5iLA(F); t2iLGö [64] PXGL [71]	mTeSR1 [44] KSR + FGF2; RSeT [64] E8 [71]
CD77	N/A	N/A (glycoprotein)	Unknown	2iL/I/F [49] 5iLA(F); t2iLGö [64] PXGL [71]	mTeSR1 [49] KSR + FGF2; RSeT [64] E8 [71]
CD7	T-cell antigen CD7	*CD7*	Potential signal transducer	5iLA(F); t2iLGö [64] PXGL [71]	KSR + FGF2; RSeT [64] E8 [71]
F11R	Junctional adhesion molecule A	*F11R*	Required for tight junction formation	5iLA(F); t2iLGö [65]	E8; RSeT, NHSM [65] (detected, but with lower signal compared to 5iLA(F) and t2iLGö cells)
SUSD2	Sushi domain-containing protein 2	*SUSD2*	Potential roles in cell adhesion, migration and signal transduction.	PXGL [71]	E8 [71]
CD320	CD320 antigen	*CD320*	Transcobalamin receptor mediating Vitamin B12 uptake	5iLA(F) [64]	KSR + FGF2 [64]

CD75 is a surface-localised carbohydrate antigen generated by sialytransferases [75]. A monoclonal antibody against CD75 shows strong and uniform reactivity to naïve hPSC (cultured in t2iLGö, PXGL and 5iLA(F)) and little reactivity to primed hPSC [64,71]. Cells generated using RSeT™ media lack CD75 expression [64]. In line with this fairly stringent expression pattern, CD75 becomes detectable only at a late stage in primed to naïve hPSC reprogramming. Characterisation of cells that are flow-sorted during reprogramming into CD75 positive or negative fractions showed that the induction of CD75 marks the cell subpopulation with the highest expression levels of naïve-associated genes and with the highest naïve colony initiating capability [64]. CD75 is detected in most cell types of the human blastocyst, suggesting that CD75 expression at this stage of development is not restricted to the pluripotent epiblast cells [64]. CD75 was once described as encoding the cell surface sialytransferase ST6GAL1 [76], and several gene databases still contain this outdated information, however a later study showed that CD75 is not a sialytransferase but instead is a glycoprotein [75]. Although unable to infer CD75 expression from transcriptional information alone, there is evidence that sialytransferases might be more active in naïve hPSC and in preimplantation embryos. For example, the sialytransferase *ST6GAL1* is highly expressed in human morula and blastocyst embryos [71] and the *ST6GAL1* gene forms a 3D chromatin interaction with a distal super-enhancer in naïve hPSC [77]. Interestingly, the super-enhancer contains many SVA-LTR5Hs repeats that are activated preferentially in naïve hPSC, and the forced repression of these repeats causes a reduction in *ST6GAL1* expression [77]. The control of sialytransferase expression and potentially their glycoprotein products including CD75 are, therefore, integrated within the regulatory pathways of naïve hPSC.

A second informative cell surface marker is CD130, which is expressed in t2iLGö, PXGL, 5iLA(F) and 3iL naïve hPSC, but not in primed cells or in RSeT-cultured cells [44,64,71]. CD130 expression is induced fairly early in primed to naïve hPSC reprogramming, and marks a broad population of cells of which only a subset of cells is also CD75 positive [64]. As a consequence, CD130 is most informative when used in combination with other cell surface markers. CD130 is expressed in the inner cell mass of human blastocysts [64], and this expression is sustained when the embryo is treated with 5iLAF to hold the cells in a preimplantation state [78]. CD130 (encoded by *IL6ST*) functions as a subunit of cytokine receptor complexes and transduces signals including leukaemia inhibitory factor (LIF), interleukin 6 (IL6), ciliary neurotrophic factor (CNTF) and oncostatin M (OSM). Interactions between the cytokine receptor and ligand leads to the activation of JAK proteins and downstream pathways such as STAT3, PI3K and RAS. Treating 3iL cells with a JAK inhibitor leads to a reduction in colony size and number, and a downregulation in the expression of predicted STAT3 target genes such as the pluripotency factor *KLF4* [44]. These observations provide important evidence for a functional role of cytokine signalling in naïve hPSC and a detailed characterisation of these pathways would be valuable to understand the signalling requirements and downstream effectors of these cell types.

CD77 is a neutral glycolipid (globotriaosylceramide) that is expressed on the surface of naïve hPSC (t2iLGö, PXGL, 5iLA(F) and 2iL/I/F) but not in primed hPSC or RSeT-cultured cells [49,64,71]. A monoclonal antibody that detects CD77 generates a broad range in fluorescent intensity signal when naïve cultures are examined by flow cytometry. CD77 helps to identify the nascent naïve hPSC during reprogramming, but is not essential as the CD77 antibody can be omitted if other naïve-specific markers are used [64]. The CD77 molecule is generated by the linkage of galactose to lactosylceramide catalysed by the enzyme A4GALT. Interestingly, CD77 expression is actively regulated on the cell surface of B lymphoma cells in response to several mitogens [79], raising the possibility that the broad range of expression in naïve hPSC could be in response to the stimulation state of individual cells. It would be interesting to test if there are differences between CD77 high and low cell subpopulations.

Another cell surface protein that was identified in an antibody screen is CD7, which is a transmembrane protein that is a member of the immunoglobulin superfamily. CD7 is expressed in t2iLGö, PXGL, 5iLA(F) naïve hPSC, but not in primed or in RSeT™ cells [64,71]. An interesting observation is that CD7 is expressed in multiple naïve hPSC lines and in different conditions, but CD7 transcripts and protein are not detectable in human blastocysts [64]. This molecule is therefore induced upon the stabilisation of naïve hPSC that are generated either directly from the embryo or by cell reprogramming. One possible explanation for this difference could be due to the loss of appropriate epigenetic repression at the *CD7* locus in naïve hPSC. In other cell types, *CD7* expression is suppressed by DNA methylation and *CD7* is reactivated after treatment with the DNA demethylating agent 5-Aza-2′-deoxycytidine [80]. Naïve hPSC are DNA hypomethylated and have a global DNA methylation landscape that is distinct from the human blastocyst including the loss of methylation at some imprint control regions [51]. It is possible that *CD7* expression is repressed by DNA methylation in the blastocyst, and this repression is eroded in naïve hPSC leading to the activation of CD7 expression. If true, then CD7 expression could provide a useful biomarker to identify conditions that maintain a more faithful DNA methylation landscape. This profile would be indicated by cells that maintain CD7 suppression while expressing other naïve hPSC markers.

F11R (also known as JAM1, JAM-A or CD321) is a transmembrane protein that is localised to tight junctions in a range of cell types. Flow cytometry using a monoclonal antibody showed that F11R is expressed in naïve (t2iLGö and 5iLA(F)) and primed hPSC, with a higher signal in naïve hPSC [65,72]. This difference in signal can be used to identify nascent naïve hPSC from within a mixed population of reprogramming cell types [65]. Specifically, the cells that have the highest F11R fluorescent signal intensity, in combination with low expression of the primed marker SSEA3 and the positive expression of the shared pluripotency marker EpCAM, define a small subpopulation of cells that are at an early stage of reprogramming (~2–5% of live cells). F11R protein is localised to sites of cell contact in the inner cell mass and trophectoderm of human blastocysts [81]. Given the essential role for F11R in maintaining tight junctions, it would be interesting to know if there are any differences in the assembly or integrity of tight junctions in naïve versus primed hPSC.

Identified using transcriptional profiling, SUSD2 is a transmembrane protein that is expressed strongly and uniformly on the cell surface of naïve hPSC (t2iLGö and PXGL) and with little expression in primed hPSC [71]. SUSD2 transcripts and protein are detected in epiblast cells of the human blastocyst, and gene expression analysis of cynomolgus primate embryos shows that *SUSD2* transcripts are high in preimplantation epiblast cells and absent in postimplantation epiblast cells, thereby validating the developmental regulation of *SUSD2* expression [71]. An antibody that detects SUSD2 can be used to monitor the emergence of nascent naïve hPSC during somatic cell or primed hPSC reprogramming to a naïve state, including in live cultures [82]. This facilitates the establishment of naïve cultures and also the identification of conditions that promote the stabilisation of naïve pluripotency. Interestingly, the proportion of SUSD2+/CD24– cells is high and variable (~10–60%) during the reprogramming of different cell lines, and the majority of the cells that first activate SUSD2 still express the primed marker CD24 [71]. It would be interesting to fractionate these different cell populations and to characterise their properties. At a molecular level, SUSD2 contains several domains including Somatomedin B (SMB), an Adhesion associated domain in MUC4 and other Proteins (AMOP), a Von Willebrand factor (vWF), and a Sushi domain [83]. Proteins that have related domains have roles associated with cell adhesion, migration and signal transduction, and in colon cancer cell lines SUSD2 expression has been implicated in growth inhibition and cell cycle arrest [84]. Whether SUSD2 has a function in naïve hPSC reprogramming is currently unknown.

CD320 is expressed on the cell surface of 5i/L/FA-cultured naïve but not primed hPSC [64,71], and the protein and transcripts are present in the epiblast cells of the human blastocyst [40,64]. The expression dynamics of CD320 in naïve hPSC reprogramming has not been reported, but this is a promising marker for future studies. CD320 is a transcobalamin receptor that mediates the uptake of vitamin B12.

Several other potential cell surface markers with a higher signal in naïve compared to primed hPSC were identified in an antibody screen, including CD66, CD32, CD107b and CD229 [64]. However, this set of markers has not yet been validated partly due to the lack of suitable antibodies and partly because they were ranked lower in priority for follow up studies as some of the markers are not transcriptionally expressed in human blastocysts. Two other studies have used transcriptional profiling of naïve and primed hPSC to identify genes that can define the different pluripotent states [85]. Messmer and colleagues compared their gene list to published datasets of preimplantation embryos from mouse, cynomolgus monkey and human, revealing several conserved pathways between species and identifying a number of genes encoding cell surface markers that might serve as useful markers to discriminate between naïve and primed states [86]. This set of genes includes *ALPP*, *ALPPL2* and *HYAL4*, which are expressed transcriptionally at higher levels in naïve compared to primed hPSC. A next step will be to determine if these predicted cell surface markers show similar state-specific protein expression profiles using antibodies. In a second study, Bernardo and colleagues performed an interspecies comparison between mouse, bovine and porcine datasets to identify genes that can discriminate between naïve and primed pluripotent states [85]. They showed that the cell surface protein Gjb5 is a consistent marker of pluripotent stem cells and preimplantation epiblast cells in all three species. However, *GJB5* is lowly expressed in naïve hPSC and is not expressed in human preimplantation epiblast cells, suggesting that *GJB5* regulation is not conserved in human development. Nevertheless, these interspecies comparisons will be informative to identify conserved criteria that predict pluripotent cell identity.

## 5 Current applications of the cell surface markers

**Fig. 1:**
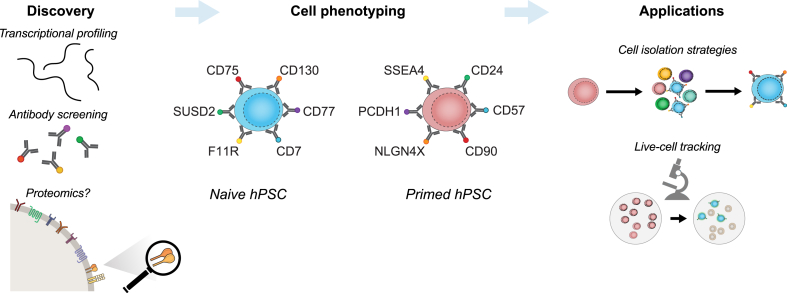
Applying cell surface markers to study human pluripotent states. Overview of the progress with applying cell surface markers and their antibodies to demarcate human pluripotent states. Studies using transcriptional profiling and flow cytometry-based antibody screens have identified informative cell surface markers. A direct proteomic measurement of plasma membrane localised proteins has not yet been reported for naïve hPSC, but this dataset would be valuable for unbiased marker discovery. The central part of the figure summarises the current set of cell surface markers that define naïve and primed hPSC. Applying the cell surface markers enables the monitoring and prospective isolation of target cell types, for instance during cell reprogramming.

The cell surface markers described above have been used primarily to compare between cells that are cultured under different conditions, thereby providing an informative benchmarking to categorise current and future cell types (Fig. 1). Naïve hPSC cultured in stringent conditions express CD75, CD77, CD130, SUSD2 and F11R-high, and lack expression of primed markers including SSEA3, SSEA4, NLGN4X and CD24. The markers have also been used to identify and isolate specific cell types such as nascent naïve hPSC out of a mixed population during somatic or primed cell reprogramming under different conditions. This approach provides an unambiguous and quantitative readout of cell state change. The isolated cells can be re-plated to establish purified naïve cell cultures, or interrogated using molecular and functional assays to define the pathways that control pluripotent states. Through these experiments, we are beginning to learn about the transcriptional dynamics that occurs during naïve hPSC reprogramming and also the differences between the cells that are reprogrammed successfully versus those that are refractory [64]. This information could help to define new molecular waypoints and to develop improved conditions that promote greater reprogramming efficiencies. One emerging picture is that different markers could provide particular advantages, for instance SUSD2 can be used as a live read out in cell culture, but other combinations of markers, particularly CD75^+^/CD130+/CD7^+^/CD24– identify a stringent population of reprogrammed cells. One of the most exciting uses of the markers is that they can be used to track specific populations of cells over time of reprogramming. Interestingly, different cell lines [71] and different reprogramming methods [[64], [65], [66]] generate nascent naïve hPSC with distinct kinetics and efficiencies. The reasons for these differences are poorly understood, but studying these processes could shed light on the trajectories that cells take during reprogramming and this information might lead to improved reprogramming efficiencies.

## 6 Conclusions and future directions

The application of cell surface marker phenotyping to resolve complex cell populations is a valuable technique to study the processes that control cell differentiation and reprogramming. There are several open questions for potential future study within this area. First, we know little about whether the cell surface markers identified in naïve hPSC are functional and also the potential mechanisms through which they might contribute. Loss of function studies using blocking antibodies or genetic perturbation should be a priority for future work. Beyond individual marker discovery, the identification of all proteins on the surface of naïve hPSC would be very valuable for investigating the pathways that control properties such as cell interactions, communication and signalling. Second, the current panel of cell surface markers could be exploited to perform large-scale screens, for instance to identify factors that are associated with the reprogramming or maintenance of naïve cultures. There are several advantages to this approach over using reporter lines, most obviously in circumventing the need to genetically alter cell lines, and the readout could be through high content flow cytometry or imaging. Third, it is clear from current data that naïve hPSC cultures are not uniform in their expression of all cell surface markers. It will be interesting to explore whether there are distinct subpopulations of cells within naïve cultures that can be fractionated using the identified surface markers. There might be functional heterogeneity or population hierarchies, similar to models that have been proposed in primed hPSC cultures. One initial way forward would be to test whether the NCAM-positive boundary cells that have been reported to exist in LIF/2i-cultured naïve hPSC hold particular characteristics or properties [27]. Similar open questions about cell heterogeneity also apply to cell populations that arise during the transitions between primed and naïve states, and single cell studies will be required to resolve these important topics. Fourth, one of the current limitations is the narrow range of suitable monoclonal antibodies that detect epitopes on naïve hPSC. The generation of additional, high-quality antibodies perhaps using a similar approach to that recently described in primed hPSC [72] would be a valuable advance. Over the next few years, the development of new reagents and the careful investigation of cell phenotype will enable the detailed characterisation of human pluripotent states. These advances will open up several exciting areas as naïve hPSC provide a unique cell model in which to test the mechanisms that underpin human developmental and stem cell biology.

## Author contribution

J.G., A.L.L. and P.J.R.-G.; writing - original draft, review and editing.
